# Randomised, double-blind, placebo-controlled trial of oral budesonide for prophylaxis of acute intestinal graft-versus-host disease after allogeneic stem cell transplantation (PROGAST)

**DOI:** 10.1186/s12876-014-0197-7

**Published:** 2014-11-26

**Authors:** Renate Schmelz, Martin Bornhäuser, Johannes Schetelig, Alexander Kiani, Uwe Platzbecker, Uta Schwanebeck, Xina Grählert, Lutz Uharek, Daniela Aust, Gustavo Baretton, Rainer Schwerdtfeger, Jochen Hampe, Roland Greinwald, Ralph Mueller, Gerhard Ehninger, Stephan Miehlke

**Affiliations:** University Hospital Carl Gustav Carus, Medical Department I, Fetscherstr. 74, 01307 Dresden, Germany; Coordinating Centre for Clinical Trials, Dresden, Germany; Charité-Campus Benjamin Franklin, Medical Department 3, Berlin, Germany; University Hospital Carl Gustav Carus, Institute for Pathology, Dresden, Germany; Deutsche Klinik für Diagnostik, KMT Zentrum, Wiesbaden, Germany; Dr. Falk Pharma GmbH, Freiburg, Germany; Center for Digestive Diseases, Cooperation of Internal Medicine, Hamburg, Germany

**Keywords:** Acute intestinal Graft-versus-host disease, Prophylaxis, Allogeneic stem cell transplantation, Budesonide

## Abstract

**Background:**

Gastrointestinal graft–versus-host disease (GvHD) is a potentially life-threatening complication after allogeneic stem cell transplantation (SCT). Since therapeutic options are still limited, a prophylactic approach seems to be warranted.

**Methods:**

In this randomised, double-blind-phase III trial, we evaluated the efficacy of budesonide in the prophylaxis of acute intestinal GvHD after SCT. The trial was registered at https://clinicaltrials.gov, number NCT00180089.

Patients were randomly assigned to receive either 3 mg capsule three times daily oral budesonide or placebo. Budesonide was applied as a capsule with pH-modified release in the terminal ileum. Study medication was administered through day 56, follow-up continued until 12 months after transplantation. If any clinical signs of acute intestinal GvHD appeared, an ileocolonoscopy with biopsy specimens was performed.

**Results:**

The crude incidence of histological or clinical stage 3–4 acute intestinal GvHD until day 100 observed in 91 (n =48 budesonide, n =43 placebo) evaluable patients was 12.5% (95% CI 3-22%) under treatment with budesonide and 14% (95% CI 4-25%) under placebo (p = 0.888). Histologic and clinical stage 3–4 intestinal GvHD after 12 months occurred in 17% (95% CI 6-28%) of patients in the budesonide group and 19% (CI 7-32%) in the placebo group (p = 0.853). Although budesonide was tolerated well, we observed a trend towards a higher rate of infectious complications in the study group (47.9% versus 30.2%, p = 0.085). The cumulative incidences at 12 months of intestinal GvHD stage >2 with death as a competing event (budesonide 20.8% vs. placebo 32.6%, p = 0.250) and the cumulative incidence of relapse (budesonide 20.8% vs. placebo 16.3%, p = 0.547) and non-relapse mortality (budesonide 28% (95% CI 15-41%) vs. placebo 30% (95% CI 15-44%), showed no significant difference within the two groups (p = 0.911). The trial closed after 94 patients were enrolled because of slow accrual. Within the limits of the final sample size, we were unable to show any benefit for the addition of budesonide to standard GvHD prophylaxis.

**Conclusions:**

Budesonide did not decrease the occurrence of intestinal GvHD in this trial. These results imply most likely that prophylactic administration of budenoside with pH-modified release in the terminal ileum is not effective.

## Background

Acute intestinal GvHD is a frequent complication after allogeneic stem cell transplantation (SCT) and remains a major cause of morbidity and mortality. In spite of standard GvHD prophylaxis between 20 to 80% of the patients suffer from clinically relevant acute GvHD [1-9]. Whereas acute GvHD with affection of the skin and/or liver rarely becomes life-threatening, acute intestinal GvHD represents one of the most frequent causes of death after allogeneic SCT. Severe cases may suffer from watery stools up to a volume of several litres, bloody stools or ileus. The median survival of correspondent patients with acute GvHD grade 3 and 4 constitute between two and three months only [8]. Therefore a prophylactic approach seems to be warranted.

Budesonide has demonstrated its efficacy in the treatment of various chronic inflammatory bowel diseases [10-20]. It is a locally acting steroid derived from 16α-hydroxyprednisolon with strong anti-inflammatory, antiexudative and anti-oedematous characteristics. The local effect of budesonide is comparable to prednisolone [11,15,21]. It underlies a high first pass effect in the liver and therefore is associated with fewer side effects compared to corticosteroids with systemic efficacy. The bioavailability accounts for 9 to 12%. Some reports of the effectiveness of budesonide and other locally acting steroids in acute GvHD already exist [22,23]. A study on the potential value of budesonide for the prophylaxis of intestinal GvHD has not been performed.

## Methods

### Study design and patients

The PROGAST trial, a study of budesonide as an agent for prevention of acute intestinal GvHD was a randomised, double-blind, placebo-controlled multicentre trial. The study was conducted at 3 centres from March 2003 through May 2007. The medical ethics committee of the TU Dresden and the ethics committee at Charité, Berlin approved the protocol, and all patients provided written informed consent. The trial was registered at https://clinicaltrials.gov, number NCT00180089.

Eligible male and female patients were at least 12 years of age and in preparation for related or unrelated allogeneic SCT. The stem cell donors –related or unrelated- were selected based on the compatibility for 10 HLA alleles (HLA-A, −B, −C, DRB1 and DQB1) by high-resolution (2 digit for class I, 4 digit for class II) DNA typing. One single allele mismatch was allowed within the same broad serotype or within a cross-reactive group. GvHD prophylaxis regimes followed international standards with cyclosporine A or tacrolimus in combination with methotrexate, optionally combined with anti-thymocyte globulin (ATG) or alemtuzumab.

Patients who received a T-cell-depleted graft, or who had received budesonide within 4 weeks prior to transplantation, as well as patients with local gut infections, apparent infectious disease, portal hypertension, profound liver function impairment, liver cirrhosis or severe psychiatric diseases were excluded.

### Study treatment and randomisation

Patients were randomly assigned to receive either oral budesonide (Budenofalk® 3 mg, Dr. Falk Pharma GmbH, Freiburg, Germany) at a daily dose of 9 mg (3 mg TID) or placebo. Budesonide was administered as a capsule. The galenical formulation assured a drug release according to a pH >6.4 which resembles the pH in the terminal ileum. Medication started one day before allogeneic SCT and was continued until day 56. Afterwards the patients went into a follow-up period until 12 months.

Randomisation was performed centrally with the use of a randomisation procedure stratified according to the relationship of the donor (related or unrelated), conditioning regimens ( dosage reduced or intensive), and in-vivo T-cell-depletion (with or without anti-thymocyte globulin (ATG)/alemtuzumab).

### Evaluation of efficacy and safety

GvHD evaluation was performed weekly starting from day 5 until day 56 after SCT and followed by visits in week 12, 16, 20, 24 and 56. Clinical signs of intestinal GvHD were classified according to Glucksberg-classification [24] of acute GvHD: occurrence of diarrhoea, bloody stools, abdominal pain, nausea and vomiting. If one of these symptoms emerged, a colonoscopy with specimens according to a standardized protocol was performed [25]. GvHD was histologically classified following Lerner’s classification [26]. Monitoring for adverse events using common toxicity criteria (CTC) was performed until 12 months after transplantation.

### Primary and secondary end points

The primary efficacy end point was the rate of patients with acute intestinal GvHD > stage 2 until day 100 after transplantation. Patients with histologic GvHD > grade 2 and patients with clinical signs of GvHD > stage 2 together with a positive histologic result for GvHD were classified as failures with respect to the primary end point. Secondary end points included the rate of patients with acute intestinal GvHD > stage 2 during follow-up until 12 months after transplantation, tolerability and safety of budesonide, severity of acute intestinal GvHD, incidence of chronic intestinal GvHD and infectious complications. Survival end points were overall and relapse-free survival, as well as relapse incidence and non-relapse mortality.

### Study oversight

The study was jointly designed by haematologists and gastroenterologists of the University Hospital Dresden. A total of three centres participated in this trial (University Hospital Dresden, Charité-Campus Benjamin Franklin in Berlin, German Hospital for Diagnostics, Wiesbaden). Data was collected and analysed by the local Coordination Centre for Clinical Trials (KKS) in Dresden. The academic authors vouch for the veracity and completeness of the data and the data analyses.

### Statistical analysis

For the primary end point, the rate of patients with acute intestinal GvHD > stage 2 after transplantation a rate of 30% for the placebo group was assumed. The expected incidence of GI GvHD seems to depend mainly on the frequency of endoscopic investigations. In fact Martin and coworkers [27] could show that the incidence of early stages of gut GvHD can be as high as 60%, especially in recipients of grafts from unrelated donors. It was calculated that 242 patients would be needed to provide a power of 80% in order to detect a difference in GvHD occurrence of 15% among the two groups (budesonide versus placebo). The software was nQuery Advisor® 7.0.

The overall incidence of acute and chronic GvHD and infectious complications were compared with the use of the chi-square test and accordingly Fisher’s exact test. Efficacy analyses were performed according to the intention-to-treat principle. All patients receiving at least one dose of the study drug were included in the safety analysis. Tolerability of budesonide was assessed by means of CTC score. The comparison of overall and relapse-free survival was made with Kaplan-Meier survival analysis [28] and adjacent log-rank test between the budesonide and the placebo group. The cumulative incidence of stage 3 to 4 GI GvHD until 12 months after transplantation was calculated with death without intestinal GVHD as a competing risk [29]. Relapse incidence and non-relapse mortality were considered as competing events. Cumulative incidences were compared with the Gray test. Cross tables were analysed by means of Fisher’s exact test.

## Results

### Patients

**Figure 1:**
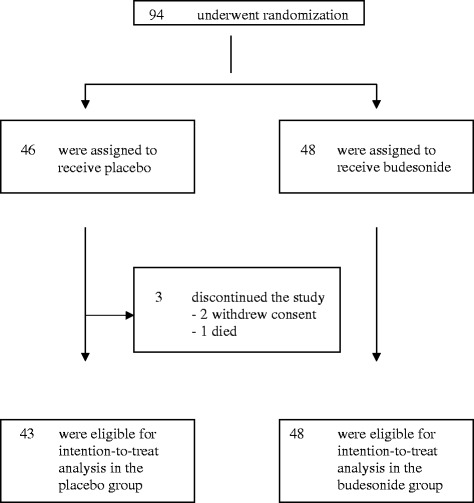
**Patient enrollment.**

Due to a lack of sufficient patient recruitment, the protocol committee decided to terminate the study prematurely. Of the 94 patients who underwent randomisation, 48 received budesonide and 46 received placebo. Three patients assigned to the placebo group did not take any study medication (2 patients withdrew their consent; 1 patient died), thus the ITT population consisted of 91 patients. The baseline disease characteristics were similar among the two groups (Table 1) except for multiple myeloma which was more frequent in the placebo group. A total of 91 patients completed the trial (Figure 1).

**Table 1:** **Demographic and clinical characteristics at baseline**

**Characteristic**	**Budesonide (N = 48)**	**Placebo (N = 43)**	**p Value**
Male sex - no. (%)	30 (62.5)	29 (67.4)	0.662
0.662 Median age (yrs) - Mean (SD)	53.1 + 12.2	52.0 + 12.9	0.660*
Duration of disease (mths) -Mean (SD)	23.4 + 33.4	15.4 + 15.4	0.139*
Complete remission pre-SZT - no. (%)	18 (38.3)	17 (40.5)	0.834
Basic disease - no. (%)			0.179
Myelodysplastic syndrome - MDS	5 (10.6)	7 (16.3)	
Acute myeloid leucemia - AML	19 (40.4)	14 (32.6)	
Acute lymphoblastic leucemia - ALL	7 (14.6)	4 (9.3)	
Chronic myeloid leucemia - CML	1 (2.1)		
Chronic lymphocytic leucemia - CLL	1 (2.1)	2 (4.7)	
Non Hodgkin lymphoma - NHL /M. Hodgkin	6 (12.8)	3 (7.0)	
Multiple myeloma	3 (6.4)	11 (25.6)	
Others	5 (10.6)	2 (4.7)	
Chemotherapeutic conditioning - no. (%)	44 (91.7)	36 (83.7)	0.246
Total body irradiation -TBI - no. (%)	27 (56.3)	22 (51.2)	0.627
Dosage reduced induction regimen (%)	27 (56.3)	24 (55.8)	0.967
In-vivo T-cell depletion (%)	29 (60.4)	26 (60.5)	0.994
Sibling transplant (%)	17 (35.4)	15 (34.9)	0.958
CMV-status positive - no. (%)	31 (64.6)	32 (74.4)	0.31

0.31P value based on a chi-square test or * t-test.

### Primary end point

Within the first 100 days after transplantation, a total of 6 patients (12.5%, 95% CI 3-22%) in the budesonide group and 6 patients (14%, 95% CI 4-25%) in the placebo group had experienced histologic or clinical acute intestinal GvHD > stage 2 according to Lerner’s- and Glucksberg classification [24,26] of intestinal GvHD. There was no significant difference within the two groups (p = 0.888, Table 2). With the final sample size of 91 patients the post hoc power to detect a 15% difference in a chi-square test was 31% (15% acute GvHD in the treatment arm compared to 30% acute GvHD in the placebo arm). The final sample size should only permit to reveal a difference of 25% (5% acute GvHD in the experimental arm and 30% acute GvHD in the placebo arm) with a type I error of 5% and a power of 80%.

**Table 2:** **Incidence of acute intestinal GvHD > grade 2 until day 100 (p = 0.888 n.s.)**

		**Group**		**Total**
		**Placebo**	**Budesonide**	
Acute intestinal GvHD grade 0-2	Number	37	42	79
	%	86.0%	87.5%	86.8%
Acute intestinal GvHD grade 3-4	Number	6	6	12
	%	14.0%	12.5%	13.2%
Total		43	48	91

Nevertheless if the trial would have continued to enroll and results continued on current trajectory there would be no significant difference (1.5%) within the two groups.

### Secondary end points

**Figure 2:**
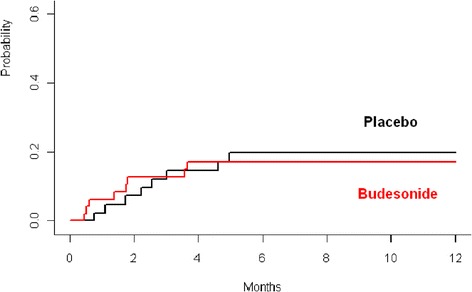
**Cumulative incidence of gastrointestinal GvHD > stage 2 after prophylaxis with budesonid and placebo (p = 0.84).**

**Figure 3:**
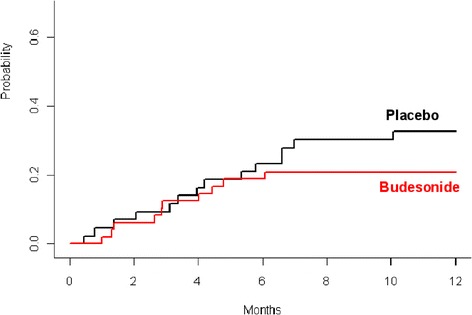
**Cumulative incidence of death after prophylaxis with budesonid and placebo (p = 0.25).**

The crude incidences of histologic and clinical stage 3–4 intestinal GvHD after 12 months observed in 91 (n =48 budesonide, n =43 placebo) evaluable patients were 17% (n =9, 95% CI 6-28%) in the budesonide group and 19% (n =8, 95% CI 7-32%) in the placebo group (p = 0.853). The cumulative incidences at 12 months of intestinal GvHD stage >2 with death as a competing event (budesonide 20.8% vs. placebo 32.6%, p = 0.250) and the cumulative incidence of relapse (budesonide 20.8% vs. placebo 16.3%, p = 0.547) and non-relapse mortality ( budesonide 28% (95% CI 15-41%) vs. placebo 30% (95% CI 15-44%), showed no significant difference within the two groups ( p = 0.911, Figures 2 and 3).

Through month 12, the incidences of adverse events (AE) and severe adverse events (SAE) were similar among the two groups (Table 3). Every patient in the budesonide group (100%) and 97.7% in the placebo group had at least one adverse event. Classified by the CTC score, budesonide was tolerated. There was a statistically insignificant trend to a higher rate of overall infectious complications until month 12 in the budesonide group (47.9%) compared to placebo (30.2%), but there was no difference in the rate of gastrointestinal infections (placebo 4.6% vs. budesonide 4.4%). Further on a statistically insignificant trend to a higher overall survival (72.9% budesonide, 62.8% placebo) in the budesonide group was observed.

**Table 3:** **Adverse events and serious adverse events**

**Variable**	**Budesonide Budesonide (N = 48)**	**Placebo (N = 43)**	**P Value**
Patients with any adverse event - no. (%)	48 (100.0)	42 (97.7)	nsa
Any serious adverse event - no. (%)	13 (27.1)	13 (30.2)	nsa
Infectious complications - no. (%)			
Adverse event	23 (47.9)	13 (30.2)	0.085 (ns)a
Pneumonia	2 (4.2)	4 (9.3)	
Sepsis	3 (6.3)	0 (0)	
Serious adverse event	5 (10.4)	1 (2.3)	0.207 (ns)b
Pneumonia	2 (4.2)	1 (2.3)	
Sepsis	2 (4.2)	0 (0)	

The incidence of chronic intestinal GvHD showed a higher percentage in the placebo group (budesonide 6.3% (n = 3), placebo 11.6% (n = 5), but failed to reach significance.

The distribution of the severity stages of acute intestinal GvHD, as well as incidence and severity stages of skin, liver and grades of overall GvHD showed no difference between the two groups.

## Discussion

GI GvHD remains a huge problem, with few therapeutic options. Acute GvHD is mediated by immunocompetent donor T cells, which migrate to lymphoid tissues soon after allogeneic stem cell transplantation. In the light of the high mortality of severe GI GvHD which often does not respond to steroid therapy, alternative treatments are being actively investigated. Besides the options of in-vivo or in-vitro T-cell-depletion, a prophylactic pharmacologic approach seems to be the most promising. Ideally, prophylactic treatment should not affect transplantation associated mortality and the incidence of relapse. T-cell-depleted grafts effectively reduce the risk of acute GvHD but are associated with a higher relapse rate because of the missing Graft-versus-leukemia effect. An intensified pharmacologic prophylaxis has been associated with an increase in the relapse rate, a higher rate of systemic infections and a late recurrence of GvHD.

A prophylactic approach with budesonide asa locally acting immunosuppressive treatment seems to be attractive because of a lower risk of systemic complications during therapy, as seen in patients with chronic inflammatory bowel disease. Underlying rationale is a strong anti-inflammatory local effect of budesonide on the one hand and a high first pass effect in the liver of more than 90%, which leads to negligible systemic effects on the other hand.

Even though the present study suggests that oral budesonide is not effective for the prevention of acute intestinal GvHD, early intervention in patients with a high-risk of gastrointestinal GvHD still seems to be an attractive strategy.

Furthermore it should be taken into account that the applied galenic formulation of budesonide has its maximum effect in the terminal ileum and right-sided colon. This is based on the pH-modified release of oral budesonide and therefore it is not adequate for prophylaxis of intestinal GvHD in the jejunum or proximal ileum. Some open-label studies showed efficacy in distal ulcerative colitis, but this finding has to be confirmed in controlled trials [30]. Therefore it remains unclear, if budesonide has a sufficient effect in the prophylaxis of GvHD in the distal colon.

Besides pharmacokinetic analyses suggest that a single dose of 9 mg budesonide may lead to a higher local concentration of budesonide compared to 3 mg three times per day [31] as performed in this study and therefore, could increase the therapeutic potential.

Overall infectious complications showed a trend to a higher frequency in the budesonide group, but gastrointestinal infections were equal in both groups and reflect better potentially side effects of locally acting budesonide.

As an additional second endpoint there was also no difference in the incidence of liver involvement, a site where the first-pass effect would lead to the assumption of a local efficacy of the study compound.

## Conclusion

In summary, this study failed to show a significant effect of prophylactic treatment with oral budesonide in preventing gastrointestinal GvHD. This study was closed prematurely because of slow accrual. Within the limitations of the sample size, no significance difference in outcomes were able to be detected in primary and secondary outcomes.
